# Influence of humor expression on suicidal ideation among adolescents: mediating effects of depressive emotion and positive emotion

**DOI:** 10.1186/s12888-020-02814-7

**Published:** 2020-08-26

**Authors:** Chun-Yang Lee, Yi-Chen Chiang, An Li, Xian Li, Yao-Ting Wu, Yu-Jung Lin, Yuchen Zhao, Xiaoke Zhang

**Affiliations:** 1 School of International Business, Xiamen University Tan Kah Kee College, Zhangzhou, China; 2 State Key Laboratory of Molecular Vaccinology and Molecular Diagnostics, School of Public Health, Xiamen University, Xiamen, China; 3 Health Bureau, Taichung City Government, Taichung, China; 4 Department of Public Health, Chung Shan Medical University, Taichung, China

**Keywords:** Adolescents, Humor expression, Depressive emotion, Positive emotion, Suicidal ideation, Mediating effects

## Abstract

**Background:**

The occurrence and degree of suicidal ideation during the past month in adolescents should be regarded seriously. Several studies have noted that humor expression style and depressive emotion may influence adolescents’ suicidal ideation. However, there is insufficient evidence concerning whether positive emotion reduces such suicidal ideation in adolescents. In addition, the relationships among humor expression, depressive emotion, positive emotion and suicidal ideation remain to be confirmed. Therefore, in this study, we aimed to test the mediating roles of depressive emotion and positive emotion in the relationship between humor expression and recent adolescent’s suicidal ideation.

**Methods:**

A total of 1551 students in junior high school completed questionnaires. The collected data were analyzed using structural equation modeling (SEM) with LISREL 8.80 and Monte Carlo resampling with R.

**Results:**

The results indicate that suicidal ideation in adolescents during the past month was related not only to humor expression but also to depressive emotion and positive emotion. The stronger the depressive emotion felt, the stronger the suicidal ideation; in contrast, the stronger the positive emotion, the weaker the suicidal ideation. Moreover, depressive emotion and positive emotion were found to mediate the relationship between humor expression and suicidal ideation; additionally, positive emotion was found to mediate the relationship between depressive emotion and suicidal ideation.

**Conclusion:**

These results highlight that depressive emotion and positive emotion may mediate the influence of humor expression on suicidal ideation among adolescents, and positive emotion may mediate the influence of depressive emotion on suicidal ideation. More attention should be paid to decreasing adolescents’ self-deprecating humor expression and depressive emotion, whereas more witty response humor expression and positive emotion should be encouraged to prevent their suicidal ideation.

## Background

Suicide is a major and serious public safety issue that can occur throughout life [1]. According to global data, approximately 800,000 people die by suicide every year. It has even become the second leading cause of death among 15–29-year-olds globally [1]. In China, suicide is the second leading cause of death among people aged 20–34 [2]. However, one of the most important risk factors for suicide is suicidal ideation [1]. One study has shown that subjects who experienced suicidal ideation at the age of 15 were nearly 12 times more likely than those that had not to have attempted suicide by the age of 30 [3].

Many factors affect suicidal ideation. In addition to the greatest risk factor, mental disorders such as depression [4–6] and anxiety [7], social skills [8] factors also influence it. Humorous expression is a social skills factor that affects suicidal ideation. A previous study has shown that both suicidal ideation and depressive emotion are negatively correlated with affiliative and self-enhancing humor styles. Among adolescents who use a more self-defeating humor, however, the risk of committing suicide is higher [9].

Adolescence is a period of transition from childhood to adulthood; adolescents not only face a high degree of competitive pressure but also experience rapid physical and mental changes; as a result, their emotions are often unstable [10]. Research shows that teenagers between the ages of 12 and 16 can express humor and can use it to solve problems in daily life and social activities [11]. However, different types of humor expression may have different effects on mental health [12]. If one cannot effectively address and adjust one’s humor style, it may lead to depression and other negative emotions and possibly even suicidal ideation. It is generally known that suicidal ideation at all ages is affected by depressive emotion [13]. Compared to adults, teenagers are more likely to be impulsive and emotionally unstable and therefore may be more likely to attempt suicide when they are very depressed or shortly after recovering from depression [14]. According to one survey, approximately 1/6 of adolescents reported serious suicidal ideation in the past year [15]. Therefore, it is necessary to help teenagers adopt appropriate types of humorous expression to cope with life events.

### The importance of humor

Humor expression are related to physical [16], mental [12, 17] and social health [18]. Among the related variables of mental health affected by humor expression, emotion [19] and suicidal ideation [9, 20] have gradually gained attention in recent years. Previous studies have suggested that humor can improve personal emotional distress and can be used as a buffer to cope with stress and adversity in daily life [21, 22]. Students with a strong sense of humor can look at problems more positively in the face of stress, making them feel less subjective stress, and they will use reevaluation and problem-solving strategies to help them reduce the threat of stress [23], thereby reducing the impact of stress events on depression. A study found that the link between neuroticism and emotional well-being is partly mediated by humor [24]. Individuals who more often use positive humor styles, such as affiliative and self-enhancing humor, have reduced melancholy tendencies and anxiety and a greater sense of happiness [25–28]. However, the more often individuals use negative humor styles such as aggressive humor and self-defeating humor, the greater their melancholy tendencies and anxiety will be; additionally, they will have lower psychological well-being [25]. Research has demonstrated that depression and anxiety are precursors of the desire to die by suicide and suicide attempts [29], while optimism is negatively correlated with suicidal ideation [30]. Perhaps this is why affiliative and self-enhancing humor styles are negatively correlated with suicidal risk; while self-defeating humor style is positively correlated with suicide risk. Individuals who naturally use self-defeating humor are at greater risk for suicidal ideation when experiencing feelings of thwarted belongingness. In contrast, an affiliative humor style may be a particularly important protective factor against suicidal ideation [9]. The cited research clearly suggests that affiliative humor can strengthen interpersonal relationships, ease social tensions, and even reduce the risk of suicidal ideation. To sum up, humor may influence suicidal ideation through depressive emotion or positive emotion. Chiang et al. found that self-deprecating, other-devaluing, body language, and witty response humor were the four main humor expression types among adolescents [31]. When the student classroom climate favored self-deprecating/other-devaluing humor, and adolescents were more accepting of such humor, adolescents engaged more often in those humor types [32].

### The importance of depressive emotion and positive emotion

Family and interpersonal stressors, negative life events, despair and depression are closely related to suicide behaviors in college students [33]. Some studies have shown that depression and suicidal ideation are highly structurally intertwined [34]. Among the risk factors of suicide, depression is important [35, 36]. Furthermore, suicidal ideation is presumed to be an attempt to relieve or reduce stress [37]. Some teenagers report that they engage in self-harm and substance abuse to “stop bad feelings” or “to get relief” [37]. The finding that suicidal ideation is positively predicted by depression provides further evidence of the recognized link between depression and suicidal ideation [38].

Optimism is often closely associated with positive emotions, which are usually measured based on the use of words such as bold, certainty, hope, optimism, pride, superior, and win [39]. Studies have shown that depressive symptoms are associated with suicidal ideation [40], and the treatment of depression is an important and achievable clinical goal that reduces suicidal ideation [41, 42]. However, another study found that after controlling for the severity of despair and depression, students with higher levels of optimism also had lower levels of suicidal ideation [43]. This may be because optimistic individuals have more positive emotions, and consequently, suicidal ideation can be reduced. This finding suggests that actively promoting positive emotions may be a valuable strategy for suicide prevention [43].

### Current study and hypotheses

**Fig. 1:**
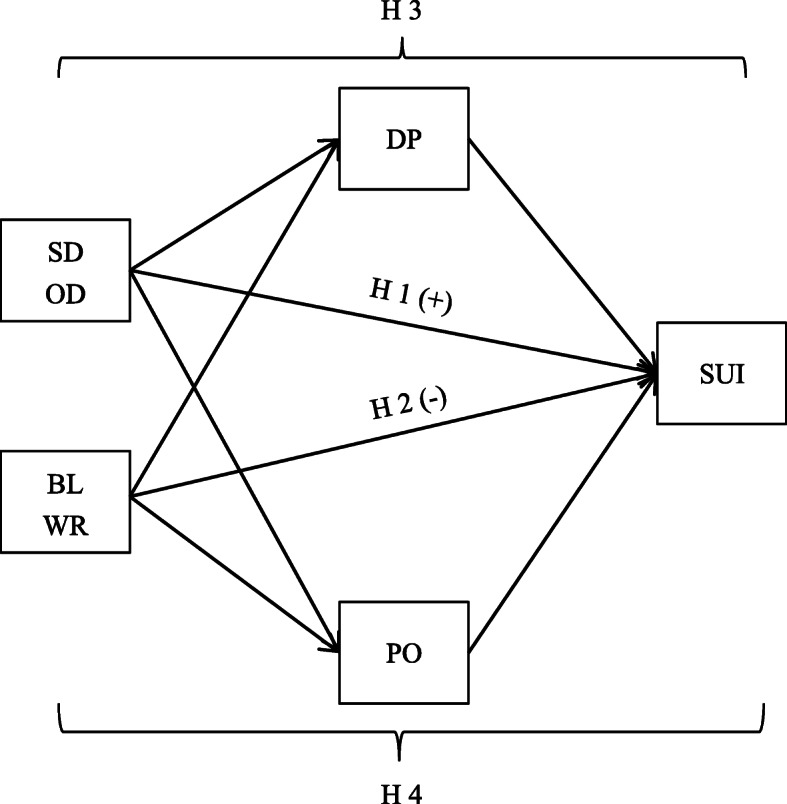
Hypothesized model of the research framework. *Note: SD:* Self-deprecating*, OD:* Other-devaluing*, BL:* Body language*, WR:* Witty response*, DP:* Depressive emotion*, PO:* Positive emotion*, SUI:* Suicidal ideation

**Fig. 2:**
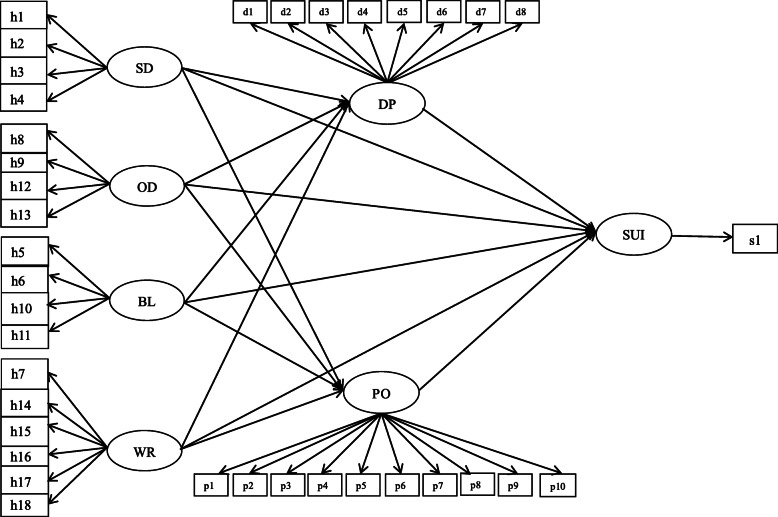
Presupposition model of the relationships among adolescents’ humor expression styles, emotions and suicidal ideation. *Note: SD:* Self-deprecating*, OD:* Other-devaluing*, BL:* Body language*, WR:* Witty response*, DP:* Depressive emotion*, PO:* Positive emotion*, SUI:* Suicidal ideation

This study aimed at investigating the correlations among humor expression, depressive emotion (DP), positive emotion (PO) and suicidal ideation (SUI) in adolescents. To this end, a research framework (Fig. 1) was constructed, a presupposition model (Fig. 2) was established, and hypotheses were proposed. We consider the self-deprecating (SD) and other-devaluing (OD) humor represent negative humor, while the body language (BL) and witty response (WR) humor represent positive humor. It was hypothesized that self-deprecating/other-devaluing humor would be positively correlated with suicidal ideation (H1). Next, it was hypothesized that body language/witty response humor would be negatively correlated with suicidal ideation (H2). Then, it was hypothesized that depressive emotion would mediate the relationship between humor expression and suicidal ideation (H3). Finally, it was hypothesized that positive emotion would mediate the relationship between humor expression and suicidal ideation (H4). To further examine the relationship between specific humor expression and suicidal ideation, we also established four subhypotheses: self-deprecating humor would be positively correlated with suicidal ideation (H1.1); other-devaluing humor would be positively correlated with suicidal ideation (H1.2); body language humor would be negatively correlated with suicidal ideation (H2.1); witty response humor would be negatively correlated with suicidal ideation (H2.2).

## Methods

### Participants and data collection

In this study, five public junior high schools were randomly selected from northern, central, southern, and eastern Taiwan. One classroom of students from each grade (seventh, eighth, and ninth grade) was randomly selected from each school. Additional details regarding the sampling procedure have been described elsewhere [32]. Overall, 1639 students completed the questionnaire, for a response rate of 95.7%. Excluding those with missing data, the valid sample totaled 1551.

In the data collection, 10 college undergraduate students or graduate students were recruited as interviewers and 10 senior interviewers as supervisors. After receiving training, they were dispatched to the sample schools to collect data. The supervisors first contacted the sample schools to obtain the schools’ consent and to confirm when the questionnaire survey would be administered. On the day of the survey, the interviewer went to the selected class in each school and had the questionnaire completed by the entire class. Prior to the administration of the survey, the participants’ parents provided informed consent. The participants completed the self-report questionnaires during class (in approximately 40 min). This study was approved by the Institutional Review Board of the College of Public Health of National Taiwan University.

### Measures

#### Humor expression types

We adopted the Taiwan Adolescent Humor Instruments (TAHI) method to measure the humor expression types; additional details regarding the 18-item scale have been described elsewhere [32]. The respondents were required to identify the extent to which they used these four different types of humor on a five-point rating scale ranging from 1 (“never”) to 5 (“always”). The Cronbach’s alphas for the self-deprecating, other-devaluing, body language, and witty response humor expression types were 0.86, 0.85, 0.86, and 0.85, respectively.

#### Depressive emotion

Adolescents self-reported their depressive emotion using the Child and Adolescent Behaviors in Long-Term Evolution (CABLE) Depression Scale. This scale was based on Kovacs’s [44] Children’s Depression Inventory (CDI) and Faulstich et al.’s [45] Center for Epidemiological Studies Depression Scale for Children (CES-DC) and is used as part of a 20-year cohort CABLE study in Taiwan every year. Depression was assessed by asking about the participant’s emotional state over the last 2 weeks (on a scale from 1 to 5, where 1 = never; 2 = seldom; 3 = sometimes; 4 = very often; and 5 = almost every day). The frequency of eight depressive emotions was evaluated: “Didn’t feel like eating favorite foods”, “Felt very sad”, “Cried for no reason”, “Found it hard to carry out tasks”, “Felt frightened”, “Didn’t sleep well”, “Lacked motivation”, and “Felt depressed”. The scores for each of the eight items were added together to give an overall total score ranging from 8 to 40. Higher scores indicated that children reported more depressive emotions. In the present sample, the depression scale was shown to have a Cronbach’s alpha of 0.88.

#### Positive emotion

Adolescents self-reported their positive emotion using the Brief Measure of Positive Affect from the Positive and Negative Affect Scale (PANAS) [46]. Studies have shown PANAS is suitable for Chinese people [47]. We conducted a back-translation procedure for the scale and confirmed the proper wording for adolescents. Students were asked about their experience of ten positive emotions, such as “excited”, “interested”, and “enthusiastic”, over the last 2 weeks and responded on a five-point scale (from 1 = not at all to 5 = very strong). The scores for each of the ten items were summed to give an overall total score ranging from 10 to 50. Higher scores indicated that children reported more positive emotions. In the present sample, the scale was shown to have a Cronbach’s alpha of 0.93.

#### Suicidal ideation

Suicidal ideation was measured by asking participants “Did you ever think of ending your own life (not wanting to live or wanting to die) during the past month?” There were 5 possible responses ranging from “never” to “almost every day”. “Have suicidal ideation during the past month” is a time category defined to identify recent high risk of suicide behavior. Therefore, we adopted “suicidal ideation during the past month” in this study to represent a high risk of future suicide behavior.

We conducted confirmatory factor analysis (CFA) for the instruments used to measure humor expression types (TAHI), depressive emotion (CABLE Depression Scale) and positive emotion (Brief Measure of PANAS). The TAHI goodness-of-fit index was as follows: (1) Chi-Square/df = 5.82; (2) RMSEA = 0.056; (3) GFI = 0.98; (4) AGFI = 0.98; (5) NNFI = 0.96; (6) CFI = 0.96. The goodness-of-fit index of the depressive emotion instrument was as follows: (1) Chi-Square/df = 9.33; (2) RMSEA = 0.073; (3) GFI = 0.99; (4) AGFI = 0.98; (5) NNFI = 0.96; (6) CFI = 0.97. The goodness-of-fit index of the positive emotion instrument was as follows: (1) Chi-Square/df = 8.34; (2) RMSEA = 0.069; (3) GFI = 0.99; (4) AGFI = 0.98; (5) NNFI = 0.96; 6) CFI = 0.97. These outcomes indicate that all the instruments have good validity.

### Data analysis

To test the hypotheses, structural equation modeling (SEM) was performed with LISREL 8.80 to analyze the relationships among humor expression, depressive emotion, positive emotion and suicidal ideation. In addition, the estimated total and indirect effects in the output file from LISREL and Monte Carlo resampling with R were adopted to confirm the significance of the indirect and total effects.

## Results

### Participant characteristics

The final sample included 802 boys (51.7%) and 749 girls (48.3%). The sex ratio was approximately 1:1. The distribution of participants across grades was similar: 30.1% of the students were in 7th grade (*n* = 467), 34.3% were in 8th grade (*n* = 532), and 35.6% were in 9th grade (*n* = 552). With respect to the area of residence, approximately a quarter of the participants were from each region. Of the different types of humor, witty response was the most common type used by students (mean = 3.25), followed by body language (mean = 2.60). Both the other-devaluing (mean = 1.88) and self-deprecating (mean = 1.85) humor types were less common. In addition, the results revealed that the students’ positive emotion (mean = 2.89) was only slightly higher than the depressive emotion (mean = 2.06), and 30.4% of the students had experienced suicidal ideation during the past month. The specific participant characteristics and descriptive statistics for the independent and dependent variables are presented in Tables 1 and 2.

**Table 1:** Participant Characteristics

Variable	n	%
Sex		
boys	802	51.7
girls	749	48.3
Grade		
7	467	30.1
8	532	34.3
9	552	35.6
Region		
northern	363	23.4
central	431	27.8
southern	404	26.0
eastern	353	22.8

**Table 2:** Descriptive statistics for the independent and dependent variables

Variable	ME	SD	n	%
*independent variables*				
Humor expression types				
self-deprecating	1.85	0.75		
other-devaluing	1.88	0.74		
body language	2.60	0.98		
witty response	3.25	1.08		
Depressive emotion	2.06	0.81		
Positive emotion	2.89	0.84		
*dependent variables*				
Suicidal ideation during the past month				
never			1080	69.6
seldom			223	14.4
sometimes to almost everyday			248	16.0

*Note: ME* Mean, *SD* Standard deviation

### The relationships among humor expression, depressive emotion, positive emotion and suicidal ideation

**Fig. 3:**
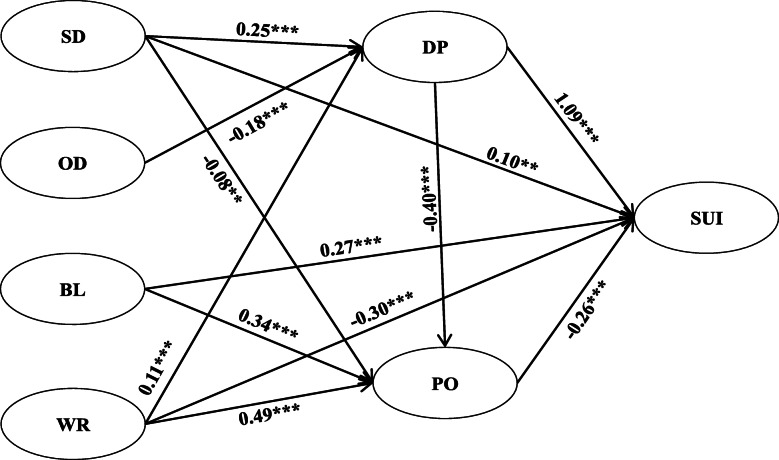
Model diagram of the mediating effect of depressive emotion and positive emotion in the association between humor expression types and suicidal ideation in adolescents. *Note: SD:* Self-deprecating*, OD:* Other-devaluing*, BL:* Body language*, WR:* Witty response*, DP:* Depressive emotion*, PO:* Positive emotion*,* SUI*:* Suicidal ideation*;* **p < 0.01; ***p < 0.001

Based on the preset model, SEM was conducted to analyze the relationships among humor expression, depressive emotion, positive emotion and suicidal ideation. It was found that some of the *t*-values were less than 1.96 in model 1. Therefore, model 2 and model 3 were obtained by deleting OD → PO, OD → SUI, respectively, according to the smallest and nonsignificant t-values. In order to explore whether there is a better model, BL → DP was deleted according to the minimum t-value to get model 4 (Fig. 3). Since the difference between the chi-square value of model 4 and model 1 (Δ*χ*^2^ = 5.82) is less than the cut-point of chi-square value (7.81) by Δdf = 3. Model 4 is our final model. The goodness-of-fit indices for each model are shown in Table 3.

**Table 3:** Comparison of the measurement models

Model	χ^2^	df	χ^2^ /df	RMSEA	NFI	GFI	NNFI (TLI)	CFI	IFI	CN
Model 1	3049.77	609	5.01	0.051	0.96	0.98	0.97	0.97	0.97	353.27
Model 2	3049.92	610	5.00	0.051	0.96	0.98	0.97	0.97	0.97	353.79
Model 3	3051.00	611	4.99	0.051	0.96	0.98	0.97	0.97	0.97	354.21
Model 4	3055.59	612	4.99	0.051	0.96	0.98	0.97	0.97	0.97	354.22

**Fig. 4:**
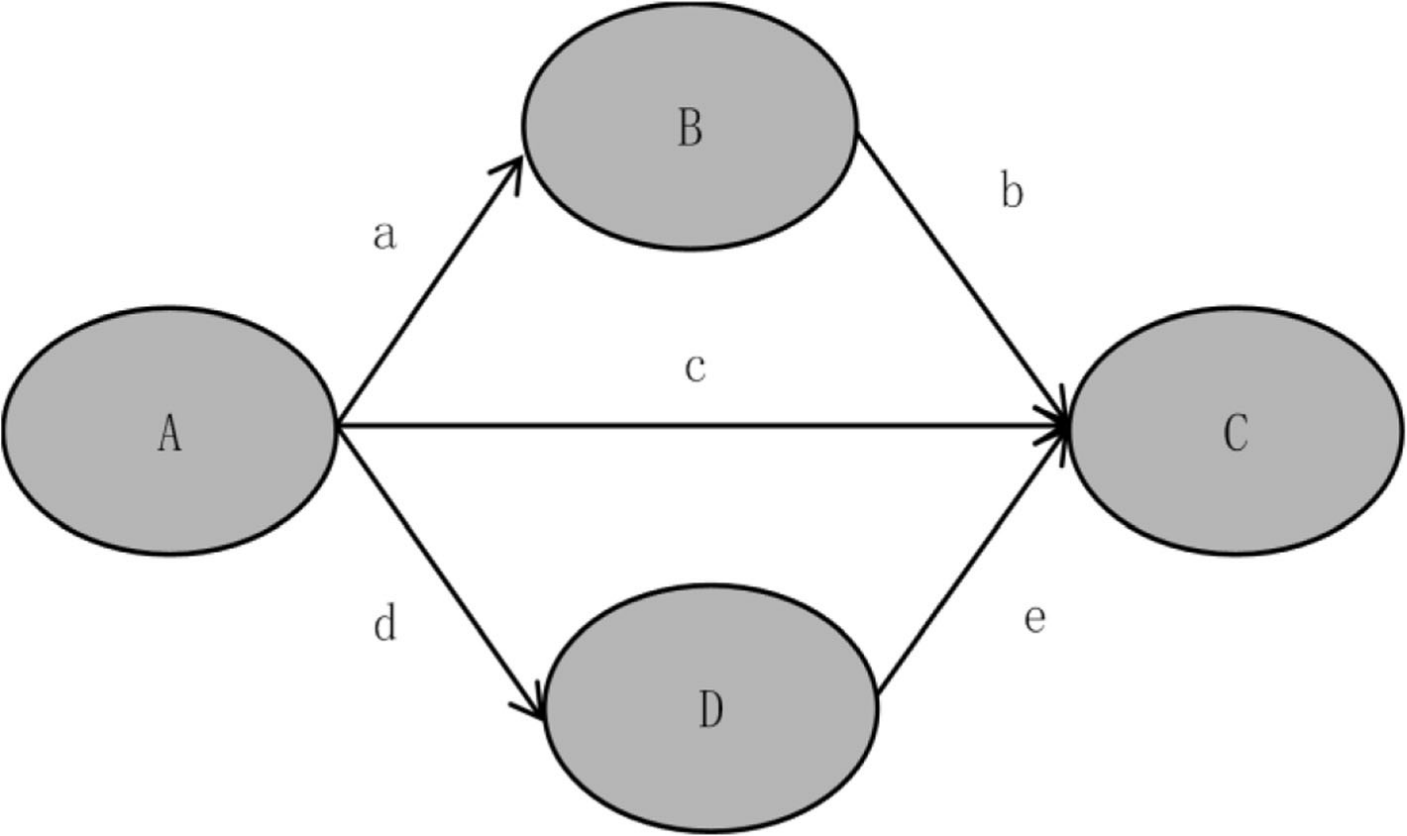
Schematic calculation diagram of direct effect, indirect effect, and total effect. The indirect effect (IE) of A on C is calculated by a *b + d * e; The direct effect (DE) of A on C is c; The total effect (TE) of A on C is calculated by IE + DE

The model diagram of the mediating effects (Fig. 3) clearly shows the direct effect among the relationships of study constructs (humor expression, depressive emotion, positive emotion and suicidal ideation). When humor expression directly acted on suicidal ideation, the effect of self-deprecating humor was significantly positive (β = 0.10, *p* < .01); H1.1 was supported; however, the relationship between other-devaluing humor and suicidal ideation lacked statistical significance; H1.2 was not supported. As with self-deprecating humor, a significant positive effect of body language humor on suicidal ideation was found (β = 0.27, *p* < .001), contradicting H2.1. In contrast, the effect of witty response humor expression on suicidal ideation was significantly negative (β = − 0.30, *p* < .001); H2.2 was supported. To sum up, H1 and H2 were partially supported. The overall direct effect, indirect effect and total effect of the humor expression on adolescent suicidal ideation were calculated by LISREL and are shown in Table 4 (the diagram and calculation formula are provided in Fig. 4). The table clearly indicates that the indirect effect of self-deprecating humor on suicidal ideation was 0.319 [0.25*1.09 + 0.25*(− 0.4)*(− 0.26) + (− 0.08)*(− 0.26)]; the indirect effect of other-devaluing humor on suicidal ideation was − 0.215 [− 0.18*1.09 + (− 0.18)*(− 0.4)*(− 0.26)]; the indirect effect of body language humor on suicidal ideation was − 0.088 [0.34*(− 0.26)]; and the indirect effect of witty response humor on suicidal ideation was 0.004 [0.11*1.09 + 0.11*(− 0.4)*(− 0.26) + 0.49*(− 0.26)]. In summary, depressive emotion mediated the relationship between self-deprecating/other-devaluing/witty response and suicidal ideation; positive emotion mediated the relationship between self-deprecating/body language/witty response and suicidal ideation. Thus, for the most part, H3 and H4 have been preliminarily supported.

**Table 4:** Descriptive statistics for the direct effect, indirect effect and total effect of the humor expression types on adolescent suicidal ideation

Path	DE	IE	TE
SD → SUI	0.100**	0.319***	0.419***
OD → SUI	–	−0.215***	−0.215***
BL → SUI	0.270***	−0.088***	0.182***
WR → SUI	−0.300***	0.004	−0.296***

*Note: SD* Self-deprecating, *OD* Other-devaluing, *BL* Body language, *WR* Witty response, *DP* Depressive emotion, *PO* Positive emotion, *SUI* Suicidal ideation, *DE* Direct effect, *IE* Indirect effect, *TE* Total effect; ^**^p < .01, ^***^p < .001

To further verify the mediation hypotheses (H3 and H4), Monte Carlo resampling was used to construct the opportune confidence intervals [48, 49]. More precisely, we used a program written in R to construct 95% confidence intervals for the indirect effects based on 20,000 resamples [48, 49]. The tests results for indirect effects in model 4 (our final model) using Monte Carlo resampling are shown in Table 5. The results reveal that the 95% confidence interval for all indirect effect in model 4 did not include zero. Therefore, H3 and H4 were partially supported.

**Table 5:** Tests of Indirect Effects of the Hypothesized Model by Monte Carlo approach of resampling

Path	95% confidence interval
SD➔DP➔SUI	[0.218, 0.327]
SD➔PO➔SUI	[0.007, 0.036]
OD➔DP➔SUI	[−0.248, − 0.145]
BL➔PO➔SUI	[− 0.129, − 0.053]
WR➔PO➔SUI	[− 0.171, − 0.087]
WR➔DP➔SUI	[0.059, 0.183]
DP➔PO➔SUI	[0.081, 0.129]

*Note: SD* Self-deprecating, *OD* Other-devaluing, *BL* Body language, *WR* Witty response, *DP* Depressive emotion, *PO* Positive emotion, *SUI* Suicidal ideation

In addition, we tried to include the three control variables (i.e., sex, grade, 3 dummy variables of area) in the final SEM model. However, no model was positive definite. To determine the influence of these control variables on suicidal ideation, we also ran SEM while controlling for the three variables using lavann with R. However, the results provided by lavann showed that the model could not be identified and that it could not invert the information matrix needed for robustness test statistics. Furthermore, we also used the proportional odds logit model to analyze control variables, independent variables and mediating variables with SAS software. The results showed that all control variables in the model had no significant relationship with the dependent variable. Hence the effect of the three control variables on the suicidal ideation is trivial.

## Discussion

The purpose of this study was to examine the mediating roles of depressive and positive mood in the relationship between humor expression and suicidal ideation in adolescents. Therefore, in addition to predicting the simple relationships among humor expression, depressive emotion, positive emotion and suicidal ideation, it was also hypothesized that depressive emotion and positive emotion would play an important role in explaining the relationship between humor expression and suicidal ideation. Studies have shown that humor expression is an important factor related to suicidal ideation; however, research has not yet examined whether depressive emotion and positive emotion can further explain this relationship. As in previous research, the results of this study revealed a relationship between humor expression and suicidal ideation as well as relationships between humor expression and depressive emotion/positive emotion. Furthermore, the current findings extend previous research by indicating that depressive emotion and positive emotion mediate the relationship between humor expression and suicidal ideation; positive emotion may be another important mediating factor between depressive emotion and suicidal ideation.

### The relationships among humor expression, depressive emotion, positive emotion and suicidal ideation

This study found that if adolescents constantly use the self-deprecation to express humor, they are likely to experience more depressive emotion [25, 50, 51] and suicidal ideation [51]. This result is consistent with research findings from other countries. We further found that teenagers had less positive emotion if they liked to use self-deprecating humor. However, adolescents who often use other-devaluing humor are less likely to experience depressive emotion [25, 26], perhaps because the object of belittlement is someone else rather than themselves. It is worth mentioning that if body language is often used to express humor, suicidal ideation and positive emotion may coexist. This result runs counter to H2.1 and H1 and may be due to the effect of adolescent hormones and the obvious increase in academic pressure in junior high school. Adolescents may tend to use reverse thinking combined with exaggerated body movements to express humor. In a similar way, Americans tend to deliberately ignore their personal sufferings through “black humor”, reflect on the awkward circumstances and encounters they face, undermine an atmosphere of terror with humor, and reveal the absurdity of the world through laughter. This style of humor showcases gloom and despair but is also a release from it that allows one to laugh at the human experience [52]. As a result, body language humor is likely to reflect suicidal ideation and the derivation of positive emotion from despair at the same time. This study originally expected that witty response humor would have a protective effect on emotion and suicidal ideation. However, the findings showed that although witty response humor was positively correlated with positive emotion and was negatively correlated with suicidal ideation, depressive emotion was still present. Perhaps, as the Mio [53] study shows, if witty response humor is used properly, it may be a helpful expression of humor, but if it is not used properly, it may have a negative effect. Therefore, the results of this study contradict the hypothesis, but whether this is because witty response humor is a more advanced form of humor requires further study.

### Mediating effects of depressive emotion and positive emotion on the effect of humor expression on suicidal ideation among adolescents

This research found that depressive emotion and positive emotion play important mediating roles in the effect of humor expression type on adolescent suicidal ideation. The adolescents who tended to use self-deprecating humor expression experienced more depressive emotions and less positive mood and had a higher degree of suicidal ideation. The indirect effect was greater than the direct effect (more than threefold), indicating that the mediating effects of depressive emotion and positive emotion were obvious and may have strengthened the influence of adolescents’ suicidal ideation. However, adolescents’ use of other-devaluing humor was not directly related to suicidal ideation. Only an indirect effect existed, which means that students who relied on other-devaluing humor experienced a lower degree of suicidal ideation with the mediating effect of depressive emotion and a positive effect. Superiority theory takes humor as arising from a feeling of superiority over others (e.g. ethnic jokes) or above one’s previous position [54]. A subset of this view is disparagement theory, which holds that humor includes ‘humorous material in which one protagonist disparages or aggresses against another one’ [55]. Humor and laughter are also considered to be useful mechanisms for easing tension in the body and protecting the body and spirit [56]. However, body language humor had a greater direct effect on adolescents’ suicidal ideation than an indirect effect through their positive emotion. In other words, adolescents who tended to use body language to express humor may have experienced less suicidal ideation in the presence of positive emotion. The indirect protective effect of positive emotion and direct protective effect of witty response humor on suicidal ideation was greater than the negative indirect effect of depressive emotion. Thus, the use of witty response humor had a negative relationship with suicidal ideation.

### Positive emotion may be another important mediating factor between depressive emotion and suicidal ideation

In the process of modifying the model goodness-of-fit indices according to the modification indices recommended by the LISREL software, it was found that depressive emotion affected the suicidal ideation of adolescents through positive emotion. This finding suggests that positive emotion may be another important mediator between depressive emotion and suicidal ideation. This finding was further confirmed by Monte Carlo resampling. Perhaps adolescents who use negative humor tend to have negative thoughts and are less likely to have positive emotion after they develop depression, which can easily result in suicidal ideation. Positive humor has the opposite effect.

### Limitations and recommendations

This study was used SEM and the Monte Carlo resampling to reveal the mediating role of depressive emotion and positive emotion in the relationship between humor expression and suicidal ideation in adolescents. However, the study’s limitations should be considered. First, this analysis is based on secondary data. We could only use single item of screening the suicidal ideation in students. However, the single item used to measure suicidal ideation in teenagers has been applied elsewhere, e.g., in a WHO international survey for school-aged children (Health behavior in school-aged children study, HBSC) [57], the American CDC Youth Risk Behavior Surveillance System (YRBSS) [58], and the 20-year cohort study “Child and Adolescent Behaviors in Long-term Evolution (CABLE) Project” in Taiwan. Addition details regarding CABLE are provided in an article we published in BMC Public Health [59]. Second, the data source used a cross-sectional research design. Therefore, the inference of causality is more conservative. As in many humor studies, it is difficult to clearly establish the time sequence of humorous expression, emotion and suicidal ideation, particularly because of the commonly used measurement duration. Third, the questionnaires used in the study were self-reported, and thus, the participants’ responses may reflect recall bias. Fortunately, our data were drawn from a representative sample from across Taiwan with a response rate of 95.7%. The findings with decent goodness-of-fit indices are worthy of scholarly attention. Furthermore, it was also suggested to test measurement invariance of our research constructs across subgroups (e.g., region and sex) of respondents.

Despite these limitations, this study provides novel information on which several recommendations for future research can be based. For example, the study suggests several strategies that can be considered in intervention programs. First, one should popularize knowledge of different humorous expression styles among adolescents to enhance their awareness of the benefits and harms of the different styles and reduce the use of harmful humorous expression styles that increase suicidal ideation. Second, one should improve the understanding of psychological guidance in adolescents and encourage them to seek help from school mental health professionals to relieve depressive and other negative emotions effectively and in a timely manner. School mental health professionals are best equipped to lead school-based suicide prevention efforts because they have training and background both in mental health and social, emotional, and behavioral strategies, as well as in multilevel systems of support [60]. Specifically, adolescents should be encouraged to help those around them create a positive and healthy state of mind. As a result, the risk of suicidal ideation and even suicidal behavior could be noticeably reduced. Third, therapists could use positive psychology interventions (PPI) to mitigate depressive emotion and to cultivate positive affect, optimism, and other positive psychological factors [61], thus reducing the risk of suicide. The results of this study confirm that in addition to depressive emotion acting as mediator between humor expression and suicidal ideation, positive emotion also plays a mediation role between humor expression and suicidal ideation. Research has confirmed that PPI effectively improves subjective and psychological well-being and reduces depressive symptoms [62]. Furthermore, there were no significant differences between internet PPI and internet cognitive behavioural therapy (iCBT) at the 6-month follow-up for depression or for happiness [63]. Both interventions are effective in reducing depression and increasing happiness. Therefore, PPI should be considered an effective way to mitigate depressive emotion and reduce suicidal ideation. Fourth, one should make full use of the clear distinguishability of different types of humorous expression to encourage people to pay close attention to and help adolescents who use negative humor (particularly self-deprecating humor) to form a protective net and reduce the possibility of adverse consequences.

## Conclusions

In conclusion, the current study reports the mediating role of depressive emotion and positive emotion in the relationship between humor expression and suicidal ideation as well as the mediating role of positive emotion in the relationship between depressive emotion and suicidal ideation among adolescents. The results of this study emphasize the importance of depression and positive emotion in adolescent humor expression and suicidal ideation. It is necessary to properly evaluate and intervene in adolescents’ depressive emotion and promote their positive emotion.

## Data Availability

The datasets used and/or analyzed during the current study are available from the corresponding author on reasonable request.

## Abbreviations

SEM: Structural Equation Modeling
DP: Depressive Emotion
PO: Positive Emotion
SUI: Suicidal Ideation
SD: Self-Deprecating
OD: Other-Devaluing
BL: Body Language
WR: Witty Response
TAHI: Taiwan Adolescent Humor Instruments
CABLE: Child and Adolescent Behaviors in Long-term Evolution
CDI: Children’s Depression Inventory
CES-DC: Center for Epidemiological Studies Depression Scale for Children
PANAS: Positive and Negative Affect Scales
CFA: Confirmatory Factor Analysis
DE: Direct Effect
IE: Indirect Effect
TE: Total Effect
HBSC: Health Behavior in School-aged Children Study
YRBSS: Youth Risk Behavior Surveillance System
PPI: Positive Psychology Interventions
iCBT: Internet Cognitive Behavioural Therapy
